# Doctor-Patient Relationship in Synchronous/Real-time Video-Consultations and In-Person Visits: An Investigation of the Perceptions of Young People with Type 1 Diabetes and Their Parents During the COVID-19 Pandemic

**DOI:** 10.1007/s12529-021-10047-5

**Published:** 2022-01-25

**Authors:** Alda Troncone, Crescenzo Cascella, Antonietta Chianese, Angela Zanfardino, Francesca Casaburo, Alessia Piscopo, Francesco Maria Rosanio, Francesca di Candia, Adriana Franzese, Dario Iafusco, Enza Mozzillo

**Affiliations:** 1 Department of Psychology, University of Campania “Luigi Vanvitelli”, Caserta, Italy; 2 Department of the Woman, of the Child and of the General and Specialized Surgery, University of Campania “Luigi Vanvitelli”, Naples, Italy; 3 Department of Translational and Medical Sciences, Section of Pediatrics, Regional Center of Pediatric Diabetes, Federico II University of Naples, Naples, Italy

**Keywords:** Type 1 diabetes, Teleconsultation, Doctor-patient relationship, Satisfaction with care, COVID-19, Physician empathy

## Abstract

**Background:**

Given that the widely acknowledged influence of the doctor-patient relationship on objective health parameters and treatment adherence in chronic illnesses, this study sought to explore how patients perceived the patient-doctor relationship across virtual and in-person contexts.

**Methods:**

Parents’ and patients’ perceptions of doctor-patient relationship were evaluated in 610 children and adolescents (12.17 ± 4.19 years, 50.9% girls) with type 1 diabetes who visited via video-conferencing or in person during the COVID-19 pandemic.

**Results:**

No differences were found between video consultations and in-person visits in terms of care satisfaction (*p* > .05), doctor-patient relationship—for the dimensions agreement on tasks (*p* = .506) and bond (*p* = .828)—as perceived by parents and physician empathy as perceived by patients (*p* = .096). Parents rated patient-doctor agreement on explicit goals of treatment higher in video consultation than in person (*p* = .009, *d* = .211). Agreement on goals (β =  − .180, *p* = .016) and bond with doctor (β =  − .160, *p* = .034) were negatively and significantly associated with HbA1c values, but only in participants who visited in person.

**Conclusions:**

Parents’ care satisfaction and perceptions of doctor-patient relationship, along with patients’ perceptions of physician empathy, did not substantially differ between visits carried out in person or via video consultations. Given the high risk of psychological problems described in young people with diabetes, video consultation can be considered a useful opportunity to maintain access to a healthcare provider in a challenging time, such as the COVID-19 pandemic.

**Supplementary Information:**

The online version contains supplementary material available at 10.1007/s12529-021-10047-5.

## Introduction

During lockdown measures for the COVID-19 pandemic, access to primary health care was seriously affected, and many non-essential services were temporarily interrupted. Additionally, in Italy, all restrictions imposed to reduce the transmission of COVID-19 led to marked decrease in clinic assistance. In particular, beginning March 9, 2020, outpatient appointments were suspended, and in-person visits were reserved only for emergencies. These measures seriously impaired the routine and general care of people affected by chronic conditions, such as patients with type 1 diabetes (T1D) [1]. Starting May 4 (after the lockdown), the Italian health system was progressively revived and restrictive isolation measures were reduced, allowing a gradual return to normality.

All over the world, the COVID-19 pandemic has forced healthcare providers to expand the use of telemedicine; thus, several diabetes telemedicine protocols and service (e.g., the use of communications technology like telephone, email, smartphone applications, computers, teleconsultation) were adopted, and telemedicine care activities were implemented by clinics worldwide in order to meet patients’ needs [2–6]. In Italy, on March 27, in line with Regione Campania’s instructions, all routine checks for patients with T1D were encouraged to be rescheduled as teleconsultation. This approach was reimbursed by the Campania Regional health system at the same rates as in-person visits.

As previously shown in research reviews on diabetes patients, telemedicine was largely considered useful support in managing diabetes and in maintaining glycemic control [7–9]. Despite some key challenges and barriers (e.g., technical problems, heavier clinician workloads) [7], the use of telemedicine has in fact been demonstrated to provide more interactions between patients and clinicians, to be an innovative and time- and cost-saving avenue of patient-clinician contact, to implement transmission and monitoring of parameters of care (e.g., levels of glycated hemoglobin, HbA1c), to support decisions in diabetes care, and to positively impact the day-to-day lives of young patients with T1D and their families [10, 11]. Accordingly, in the time of the COVID-19 pandemic, evidence from Italian studies carried out with individuals with T1D—who were monitored remotely by diabetes clinic teams during the beginning of the lockdown—indicated an improvement in several indicators of glucose control [12–14].

However, both in Italy and worldwide, studies carried out during the COVID-19 pandemic involving individuals with T1D who were followed through telemedicine have primarily explored diabetes-related variables, such as metabolic complications, general diabetes/glycemic control, and perceptions of the use and usefulness of telemedicine during the lockdown [6, 12–15]. To date, no studies have analyzed how individuals with T1D—and, for pediatric patients, their caregivers—perceive the doctor-patient relationship as experienced through telemedicine.

Given that the widely acknowledged influence of the doctor-patient relationship on objective health parameters and treatment adherence in chronic illnesses [16, 17] and in diabetes patients [18–20], the present study sought to explore how patients perceived the patient-doctor relationship across virtual and in-person contexts. Additionally, given the significant associations between parental psychological factors and children’s diabetes management [19, 21, 22] as well as between good provider-parent relationships and pediatric health outcomes [23, 24], special attention was paid to caregivers’ perceptions as well.

In particular, the aims of this study were to evaluate:

1. The perceptions of the doctor-patient relationship in young people with T1D and their caregivers during teleconsultation and in-person visits.
2. The associations between the perception of the doctor-patient relationship and glycemic control.

Analyses were adjusted for sociodemographic, anthropometric, and clinical data.

## Method

### Participants and Procedure

This is a questionnaire-based cross-sectional study.

Patients who regularly visit the Regional Referral Center for Pediatric Diabetology “G. Stoppoloni” of the A.O.U. L. Vanvitelli and the Regional Reference Center for Pediatric Diabetology of the A.O.U. Federico II were consecutively enrolled in the study with their parents if they met the selection criteria. Inclusion criteria were diagnosis of T1D, aged 10–18 years, with a primary caregiver who was literate and capable of filling out the online questionnaires. Exclusion criteria included having other illnesses (severe disability due to disease, significant comorbidity, other diagnosed diseases). Participants’ clinical records were examined to confirm that the inclusion/exclusion criteria were met.

Data were collected on outpatient visits carried out during the COVID-19 pandemic either remotely (April–May 2020) or in person (June–July 2020).

Specifically, during the lockdown, all patients for whom a diabetes in-person visit was scheduled but canceled due to the COVID-19 pandemic were contacted by phone to reschedule the visit as a video-consultation. Medical staff invited scheduled patients a few days before their appointment and informed them of the instructions to access the televisit and to help with technical issues. All video-link consultations were proposed by the hospital staff and conducted using the Webex meet platform, Skype, or Zoom. After the lockdown, the suspended treatment paths were restarted; thus, in-person visits were gradually re-added to the schedule and conducted following the assistance pathways/protocols established to protect the health of both patients and healthcare personnel.

In both scenarios (teleconsultation/in-person), patients were visited by the diabetologist; at the end of the visit, those parent caregivers participating in the visit who declared their willingness to participate in the study were sent a link on a smartphone. Caregivers were asked to sign the informed consent, to fill in the questionnaire, and to have their child with T1D fill in the questionnaire via web-based form. Participants were evaluated only once. No compensation or incentives were given for participation.

The research team—a diabetologist, a resident, a psychologist, a dietician, and a nurse—collected the information of all the participants that was necessary for the study in a strictly anonymous form; participants were identified according to an alphanumeric code.

### Measures

Sociodemographic, anthropometric, and clinical/metabolic data were collected through consultation of the electronic medical records. For each patient, the following data were recorded by the doctor on a specific information sheet: age, gender, height, weight, disease duration, multiple daily injections (MDI)/continuous insulin infusion (CSII) insulin therapy, type of sensor device (if used), recent infection with COVID-19 (if any), and glycemic control. For patients scheduled to be visited via video consultation who used a glucose sensor—whether continuous glycemic monitoring (CGM) or flash glucose monitoring (FGM)—a current HbA1c values estimation was obtained from the CGM/FGM mean glucose values of the one-month period prior (the previous 4 weeks), in line with evidence that at least 14 days of CGM data is a good estimation of HbA1c values [25]. Estimated HbA1c values were calculated according to ADAG (A1C-derived average glucose) study group data [26].

The evaluation of the doctor-patient relationship was carried out by individual administration of the following questionnaires.

#### Physician Empathy

The Jefferson scale of patient perceptions of physician empathy (JSPPPE) [27] is a 5-item self-report questionnaire measuring the patient’s evaluation of the level of empathy shown by the doctor. Patients rate physician’s empathy on a 5-point Likert-type scale (from 1 = strongly disagree to 5 = strongly agree), with higher scores indicating higher levels of empathy. Studies on the psychometric properties of the JSPPPE have reported good psychometric characteristics [28, 29].

#### Satisfaction of the Quality of the Care (Doctor’s Performance)

Comprehensive assessment of satisfaction with care (CASC) [30] is a self-report questionnaire designed to assess patients’ perceptions of and satisfaction with the quality of the care they receive. The present study adopted the CASC subscale on doctors’ behavior, which is composed of 19 items evaluating the patient’s satisfaction with the doctor’s performance across 4 dimensions. These dimensions are availability (5 items: frequency of visits, ease of obtaining a consultation, coordination among doctors, coordination between doctors and nurses, time spent with patient), interpersonal skills (6 items: questions on all difficulties, listening, interest in the person, support, human qualities, information on resources), technical skills (5 items: physical assessment, physical examination, attention to earlier health, understanding of illness, treatment/follow-up), and information provision (3 items: information on illness, information on treatment, information on tests). Patients are asked to rate each item from “poor” to “excellent” and to mention whether they want improvement (yes/no) on that dimension. Higher scores indicate higher satisfaction. Studies on the psychometric properties of the CASC have shown a high level of internal consistency and convergent validity in many countries, including in Italian samples [31].

#### Alliance in the Doctor-Patient Relationship

The Working Alliance Inventory short form (WAI-S, observer version) [32] is a 12-item questionnaire assessing alliance in the doctor-patient relationship as perceived by an external observer. Raters were asked to respond on a 7-point Likert-type scale to items across three dimensions of the doctor-patient relationship: agreement on goals (the extent to which patient and doctor agree on explicit goals of treatment), agreement on tasks (the extent to which patient and doctor agree on explicit tasks needed to achieve the goals of treatment), and bond (the extent to which patient and doctor form an emotional bond that is characterized by mutual trust and liking). In line with the characteristics of this study and with previous research [33], where necessary, the wording of some items was modified (e.g., use of the term “diabetologist” instead of “doctor/therapist”). A higher score indicates greater alliance. The WAI, along with the observer version, is used internationally to measure working alliance and has been demonstrated to have good psychometric properties [34–36].

For the present study, the validated Italian versions of the measures were used (CASC [31]; JSPPPE [37]; WAI-s [38]). Children older than 10 years were asked to fill out the JSPPPE, while parents were asked to fill out the CASC and the WAI. In both the CASC and the WAI, all items were used to evaluate parents’ opinion on how they perceive the relationship between their son/daughter and the doctor.

### Statistical Analysis

The sample size was calculated using the formula *n* = *Z*^2^ *pq*/*d*^2^, where *Z* is the standard estimate (1.96), *q* = 1-*p*, and *d* is precision at 0.05 [39]. The *p* estimation (the expected value of the population prevalence rate) was based on the higher prevalence of telemedicine satisfaction among parents of youths with T1D (approximately 80%) found in previous studies [8, 40]. Thus, the calculated sample size was 246 for each group. However, with an eye to potential dropout, more participants than the calculated number were enrolled.

Cronbach’s alpha (*α*) was computed to assess the homogeneity of the scales. Results were reported in mean ± standard deviation or in absolute and relative frequencies. The relationships among categorical variables were analyzed using chi-square contingency tables. The comparison of continuous variables between the video and the in-person groups were analyzed with Student’s *t*-tests. An analysis of covariance (ANCOVA) was used to compare groups while controlling for potential confounding variables. Results were considered significant at alpha = 0.05 for a two-sided test.

Hierarchical multiple regression analyses, with three blocks entered, were conducted to evaluate the relationship between doctor-patient relationship perception and glycemic control. For both groups, the dependent variables were parents’ satisfaction with care (CASC subscale scores), parents’ perception of alliance in the doctor-patient relationship (WAI-S subscale scores), and patients’ perceptions of physician empathy (JSPPPE score). Age, gender, illness duration, and zBMI were entered in step 1 of the regression as control variables; Hba1c was entered in step 2; and CASC/WAI-S/JSPPPE scores were entered in step 3 (depending on the dependent variable). In addition, the predictive contribution of patients’ perceptions was considered in the analysis of parents’ perceptions of doctor-patient relationship, and vice versa. Regression analyses were performed separately for video-consultation and for in-person visits. Tolerance values of > 0.1 were considered acceptable, to exclude multicollinearity [41]. Video-conferencing participants not using a CGM/FGM device were excluded from mean comparisons of glycemic control and the hierarchical multiple regression, due to missing data on current estimated HbA1c values.

The statistical analysis was performed with Statistical Package for the Social Sciences (SPSS) version 25.0 for Macintosh.

## Results

### Sample Characteristics

A total of 397 patients were contacted to reschedule appointments as teleconsultation in the period April–May 2020. Initially, *N* = 10 declined, but they were shortly rescheduled according to the patient’s requests. Out of 397 video consultations, only *N* = 79 (about 20%) were not successfully completed—mostly due to the patient/caregiver’s inability to deal with the hardware and/or software or broadband connection failure—but these were rescheduled in the days after, and support for successful access to video consultation was provided.

All 397 parents were asked whether they would be willing to participate in the study; 314 parents agreed, and 83 (approximately 20.1%) did not (*N* = 17 for general worries that their children would undergo psychological evaluation; *N* = 29 for reluctance/difficulty in using mobile phones and web-based information, *N* = 37 lack of interest). After a second analysis, *N* = 9 participants were excluded from the sample (*N* = 314) since they did not meet the inclusion criteria (*N* = 6 without T1D; *N* = 3 older than 18 years). The sample for the video consultation group comprised 305 participants (*N* = 216 using a CGM/FGM device).

Out of 502 routine outpatient visits scheduled in the period June–July 2020, only *N* = 9 missed their appointment due to personal difficulties. Recruitment ended after the target sample size was satisfied. All 493 parents who visited in person were invited to join the study: 342 parents agreed to participate, 151 (approximately 30.6%) did not (*N* = 6 their children did not have T1D, *N* = 31 could not be reached by phone due to lack of answers, *N* = 7 declined to participate because unavailable due to being busy with other activities, *N* = 107 lack of interest). Out of the 342 parents who agreed, 305 participants who best matched the video-consultation group for age and gender were selected.

The final sample was composed of 610 participants. General characteristics of the participants are shown in Table 1.

**Table 1:** Sociodemographic, anthropometric, and clinical characteristics of study subjects, compared across video consultations and in-person visits

	Video-consultation *N* = 305	In person *N* = 305	*p*	Effect size
Males (%)	52	46	.124	*V* = .062
Age (years)	12.17 (4.19)	12.12 (4.17)	.879	*d* = .012
Duration of diabetes (years)	4.27(3.88)	4.93 (3.81)	.037	*d* = .172
zBMI	.24 (5.43)	.81 (1.06)	.071	*d* = .146
HbA1c (%)	7.46 (1.04)^a^	7.63 (1.46)	.126	*d* = .134
CSII (%)	27.9	13.1	≤ .0001	*V* = .184
Using sensor CGM/FGM (%)	18.36/52.46	14.43/61.64	.07	*V* = .084
Infected with COVID-19 (%)	6.6	5.7	.625	*V* = .020

Data are means ± SD unless otherwise stated

*zBMI* standardized body mass index, *HbA1c* glycated hemoglobin, *CSII* continuous insulin infusion, *CGM* continuous glycemic monitoring, *FGM* flash glucose monitoring

^a^Values as estimated in participants using CGM/FGM (*N* = 216)

No statistically significant differences were found between the two groups (video consultation/in-person) in terms of distribution of gender, age, glycemic control, zBMI, CGM/FGM use, or COVID infection frequency (all *p* > 0.05); patients who visited in person showed a longer duration of illness (*p* = 0.037, *d* = 0.172) and lower frequency of use of insulin pump (*p* ≤ 0.0001, *V* = 0.184) (Table 1) compared to those who visited via video consultation.

The non-participating patients (video consultation *N* = 83; in-person visit *N* = 151) did not differ from those who participated in terms of distribution of gender (video: 47 m/36 f; in person: 84 m/67 f), mean (± SD) age (video: 12.29 ± 3.87; in person: 12.62 ± 3.58), duration of illness (video: 5.05 ± 3.93; in person: 5.39 ± 3.88), HbA1c (video: 7.67 ± 0.71; in person: 7.76 ± 1.52), zBMI (video: 0.58 ± 1.01; in person: 0.74 ± 1.08), insulin therapy (video: 65MDI/18CSII; in person: 136MDI/15CSII), sensor CGM-FGM/no sensor use (video: 23–41/19; in person: 20–97/34), or COVID infection/non-infection (video: 6/77; in person: 16/135) (video: *m*/*f p* = 0.467; age *p* = 0.833; duration of illness *p* = 0.108; estimated HbA1c *p* = . 108; zBMI *p* = 0.565; MDI/CSII, *p* = 0.252; sensor CGM-FGM/no sensor, *p* = 0.146; infected/not infected with COVID, *p* = 0.714; in-person visit: *m*/*f p* = 0.050; age *p* = 0.185; duration of illness *p* = 0.229; HbA1c *p* = 0.386; zBMI *p* = 0.505; MDI/CSII, *p* = 0.315; sensor CGM-FGM/no sensor, *p* = 0.862; infected/not infected with COVID, *p* = 0.140).

### Doctor-Patient Relationship Perception

The measures of parents’ perceptions of and satisfaction with the quality of the care (CASC: Cronbach’s alpha = 0.973), their perceptions of alliance in the doctor-patient relationship (WAI-S: Cronbach’s alpha = 0.825), and patients’ perceptions of physician empathy (JSPPPE: Cronbach’s alpha = 0.896) demonstrated good internal consistency.

No differences were found between video consultation and in-person participants regarding care satisfaction dimensions (doctor’s availability, interpersonal skills, technical skills, information provision; all *p* > 0.05), nor in doctor-patient relationship for the dimension agreement on tasks (*p* = 0.506) and bond (*p* = 0.828). Parents evaluated patient-doctor agreement on explicit goals of treatment higher in video consultations than in person (*p* = 0.009, *d* = 0.211). No differences were found between video consultations and in-person visits in physician empathy as perceived by patients (*p* = 0.096) (Table 2). In a replication of the analysis with duration of illness and type of insulin therapy as additional covariates—since these were the only clinical variables in which the video and in-person groups showed a significant difference (Table 1)—all the results described above were confirmed (CASC: availability *F*(3,607) = 0.087, *p* = 0.768; Interpersonal skills *F*(3, 607) = 0.398, *p* = 0.528; technical skills *F*(3, 607) = 0.813, *p* = 0.367; Information provision *F*(3,607) = 0.988, *p* = 0.321; WAI-S: agreement on goals *F*(3,607) = 5.069, *p* = 0.025, *η*^2^ = 0.008; agreement on tasks *F*(3, 607) = 0.110, *p* = 0.741; bond *F*(3,607) = 0.068, *p* = 0.795; JSPPPE: *F*(3, 400) = 2.218, *p* = 0.137).

**Table 2:** Parents’ care satisfaction (CASC), parents’ perceived alliance in the doctor-patient relationship (WAI-S), and patients’ perceptions of physician empathy (JSPPE), compared across video consultations and in-person visits

	**Video-consultation** ***N* = 305**	**In person** ***N* = 305**	***P***	**Effect size** ***d***
	**Mean (SD)**	**Mean (SD)**		
	***N* = 305**	***N* = 305**		
CASC availability	3.85 (.97)	3.83 (.92)	.837	.021
CASC interpersonal skills	3.96 (.95)	3.97 (.91)	.868	.011
CASC technical skills	4.17 (.85)	4.19 (.77)	.689	.025
CASC information provision	4.11 (.91)	4.14 (.85)	.635	.034
	***N* = 305**	***N* = 305**		
WAI-S agreement on goals	23.08 (4.66)	22.07 (4.89)	.009	.211
WAI-S agreement on tasks	25.37 (3.24)	25.19 (3.33)	.506	.055
WAI-S bond	24.99 (3.94)	24.92 (3.88)	.828	.018
	***N* = 221**	***N* = 180**		
JSPPPE	28.92 (6.03)	27.82 (7.21)	.096	.165

Data are means ± SD unless otherwise stated

*CASC* comprehensive assessment of satisfaction with care, *WAI-S* Working Alliance Inventory short form, *JSPPPE* Jefferson scale of patient perceptions of physician empathy

With regard to the aspects of care in which they would like to see improvements, parents more frequently deemed all levels as not needing to be improved, with no differences between video consultations and in-person visits in each of the areas questioned (all *p* > 0.05) (Table 3).

**Table 3:** Aspects of care (as described in CASC) deemed by parents to be improved (or not), compared across video consultations and in-person visits

CASC—“I would like to see improvement at this level”	Video-consultation *N* = 305	In person *N* = 305	*p*	Effect size *V*
	*% no*	*% no*		
1. The way they carried out your child’s physical examination	82	83	.831	.009
2. The attention they paid to your son/daughter’s previous state of health	85^a^	84	.589	.022
3. The understanding they have of your child’s illness	87	87	1.00	.000
4. The treatment and medical follow-up that they have planned	83	81	.597	.021
5. The questions they asked you about your child’s physical problems	84	84	.825	.009
6. The questions they asked you about your child’s difficulties in general (personal, social, scholastic, familiar…)	76	75	.707	.015
7. Their willingness to listen to all your child’s concerns	84	80	.292	.043
8. The information they gave you concerning your child’s illness	84	82	.453	.030
9. The information they gave you concerning your child’s medical tests	80	82	.605	.021
10. The information they gave you concerning your child’s treatment	85	87	.642	.019
11. The information they gave you about the kind of help available (psychological help, nutritional consultation, oculist visit…)	72	78	.091	.069
12. The interest they showed in your child personally and not just in his/her illness	82	82	.833	.009
13. The comfort and support they gave your child	83	81	.597	.021
14. Their human qualities (politeness, respect, sensitivity, kindness, patience,…)	89	88	.701	.016
15. The number of their visits/consultations	81	83	.674	.017
16. The time they devoted to your child during their visits/consultations	81	85	.234	.048
17. The ease of obtaining an interview with a doctor (diabetologist)	77	78	.697	.016
18. The coordination between the doctors	84	84	.912	.004
19. The coordination between the doctors and nurses	85	85	.821	.009

*CASC* comprehensive assessment of satisfaction with care

^a^These totals do not correspond to 305 due to missing answers

### Doctor-Patient Relationship Perception and Diabetes Control

The results of the regression analyses are reported in Supplemental Tables 4, 5 and 6. HbA1c values (entered at step 2 of the regression analyses) accounted for a significant amount of variance in parents’ perceptions of doctor-patient alliance in in-person visit participants (WAI-s *ΔR*^2^ range = 0.025–0.031, all *p* < 0.05), but not in video-consultation participants (CASC *ΔR*^2^ range = 0.00–0.002, all *p* < 0.05; WAI-S *ΔR*^2^ range = 0.00–0.012, all *p* > 0.05) (Supplemental Tables 4, 5). In addition, duration of illness and gender were found to be significantly associated with parents’ perception of doctor-patient alliance, but only in participants seen in person (*p* range = 0.01–0.036) although most cases within models with low predicting power (Supplemental Table 5).

Patient’s perception of physician empathy (entered in step 3 of the regression analyses) significantly increased parents’ satisfaction with care and perception of doctor-patient alliance variance in both in-person visit participants (CASC: *ΔR*^2^ range = 0.291–0.453, all *p* ≤ 0.0001); WAI-S: *ΔR*^2^ range = 0.098–0.466, all *p* ≤ 0.0001) and video-consultation participants (CASC: *ΔR*^2^ range = 0.271–0.418, all *p* ≤ 0.0001; WAI-S: *ΔR*^2^ range = 0.109–0.496, all *p* ≤ 0.001) (Supplemental Tables 4, 5). Similarly, parents’ satisfaction with care and perception of doctor-patient alliance (entered in step 3) accounted for a significant amount of variance in patients’ perception of physician empathy variance in both video-consultation (*ΔR*^2^ = 0.597, *p* ≤ 0.0001) and in-person visit (*ΔR*^2^ = . 584, *p* ≤ 0.0001) participants (Supplemental Table 6).

## Discussion

The present study aimed to contribute to the knowledge on possible characteristics of and differences in patients’ and parents’ perceptions of patient-doctor interactions across virtual and in-person contexts. As far as we know, this study is the first to evaluate the doctor-patient relationship as perceived in video consultations vs. in-person visits for pediatric T1D patients during the COVID-19 pandemic. It was also innovative in its specific focus on caregivers’ perception—which, due to parents’ involvement in decisions about their child’s care, was considered essential in order to fully address this study’s aims.

In its attempt to extend previous research on the usefulness of and satisfaction with telemedicine in diabetes management—especially in relation to studies producing mixed results [42, 43]—this study provides empirical evidence that for T1D patients and visits via video consultation were not inferior to those in person. According to the present results, parents showed a generally high level of satisfaction with care provided via both video consultation and in-person visits. Specifically, in spite of the lack of in-person care, the use of video consultation was found to not be characterized by a different perception of the doctor-patient relationship: parents’ care satisfaction and doctor-patient relationship perceptions, as well as patients’ perceptions of physician empathy, did not differ between visits carried out remotely or in person. Additionally, it should be noted that one of the dimensions of the doctor-patient relationship analyzed here—patient-doctor agreement on explicit goals of treatment—was perceived by parents as even higher in video consultations than in in-person visits.

Taken together, these results can be considered to be in line with previous evidence describing the use of telemedicine (i.e., videoconferencing, phone contacts, remote glucose downloads, and web-based education systems) as a safe and effective way to provide diabetes care [9] [44–46], and as a particularly helpful approach in improving positive long-term outcomes and general well-being [47]. It is also in line with recent studies on Italian T1D patients who were followed remotely during lockdown and who were described as actually improving in their glycemic control [12–14].

Moreover, while participants who visited remotely showed no significant associations between parents’ patient-doctor relationship perceptions and glycemic control, in the case of participants who visited in person, parents’ perceptions of the doctor-patient alliance were negatively associated with HbA1c values and also, albeit weakly, correlated with gender and duration of illness. The association between parents’ and patients’ perceptions of doctor-patient variables was similar in both the virtual and the in-person settings, suggesting a reciprocal relationship or perhaps reciprocal reinforcement between caregivers’ and patients’ opinions, regardless of the context.

With regard to the higher agreement on goals in the video consultation group, it could be hypothesized that, in this context, difficulties related to the pandemic as well as limitations imposed by the lack of physical contact might have further focused patients’ and doctors’ attention on mutual objectives. Difficulties due to the pandemic and the lack of alternatives to video consultations might have contributed to parental perceptions, in that they appreciated the opportunity for their child to be seen regardless of COVID-19-related constraints and thus experienced a patient-doctor relationship that primarily focused on what the visit could provide them and their child in order to obtain answers to their primary needs and concerns.

We can also speculate that, for patients who visited in person, glycemic control values might have played a role in the patient-doctor relationship, so that lower glycemic control was associated with lower perceptions of a therapeutic alliance for this group. Despite the fact that HbA1c values informed diabetes management during the previous months, these values might have been considered by parents as a current indicator of unsuccessful diabetes management, which in turn may have contributed to how parents perceived the relationship with the doctor at the time. These suppositions can be supported by previous evidence that describes the diabetic visit—especially the occasion of receiving HbA1c results—as a source of anxiety and worries for caregivers and for children, and poor blood test can be perceived by the caregivers as a reflection of their ability to succeed in their child’s diabetes care [48]. Similarly, other studies have highlighted significant and more general associations between doctor-patient relationships aspects and glycemic control [49, 50].

However, an interpretation of the differences in the associations between perceived doctor-patient alliance and glycemic control across teleconsultations/in-person visits requires taking some general issues into account.

First, since regression analyses were conducted separately for teleconsultation and in-person visit participants, it cannot be determined whether the group differences in the associations between WAIS scales and HbA1c values were statistically different. Second, since not all patients in the video consultation group used FGM/GCM, the analysis sample for the relevant regression analyses was smaller than that for the in-person group; this might have reduced the power to detect a significant relationship between WAIS/CASC/JSPPE scores and glycemic control. Third, HbA1c was measured using two different methods in both groups, so it cannot be ruled out that the different associations observed in the regression analyses might be due to the different measurement methods of HbA1c. As a result of all these, the differences in the associations between WAIS scales and HbA1c values in both groups should be cautiously interpreted.

Overall, although we agree with Noorgard [51] that not all consultations should be carried out via video-link consultations and that in-person contact between patients and doctors is invaluable for chronic disease patients with T1D and their caregivers, video consultation was confirmed in the present study as a useful opportunity to maintain access to a healthcare provider. Given the high risk of psychological problems described in young people with T1D [52–56], protecting the interaction between patients and their doctors, even if said interaction is provided remotely, appears particularly important—especially in such challenging times as the COVID-19 pandemic [57].

However, some limitations should be considered when interpreting the present findings, particularly the sample selection bias (participation done on voluntary basis, all participants from the same geographic area, relative high non-participation rate, non-random selection of participants) and the imprecise ratings of clinical and psychological data (e.g., use of psychological self-reported measures). In particular, due to differences in the methods of collecting HbA1c values (i.e., estimated HbA1c for video consultations vs. direct measurement of patients’ blood for in-person visits), a part of the sample could not be included in the analyses, meaning that glycemic control-related results and observations should be viewed with extra caution. Moreover, the lack of assessments of the time ranges from the CGM/FGM and of information about the incidence of hypoglycemic and hyperglycemic events, as well as the use of glycosylated hemoglobin as a unique measure of glycemic compensation, may have hindered full comprehension of possible differences in diabetes control. Furthermore, findings concerning patients’ perceptions of physician empathy must be interpreted cautiously, because to date, JSPPPE is a commonly used and validated measure among adults. Moreover, the cross-sectional study design made direct comparison between video and in-person consultations difficult and prevented any conclusions on causal connections in the significant associations.

In conclusion, with regard to the possible clinical implications, the present results are in agreement with previous research [2, 11] and suggest that telemedicine care can become an integral part of healthcare delivery—especially for chronic illnesses like T1D—that may replace some outpatient appointments as an add-on service. At the same time, the present findings underscore that greater awareness and careful attention should be paid to the patient and parent perspectives, especially when the doctor-patient relationship takes place in different contexts. In order to meet patients’ needs and to ensure the success of diabetes care, diabetes healthcare providers should always be mindful of the extent to which their behaviors may contribute to patients’ and caregivers’ perceptions of the doctor-patient relationship—particularly when dealing with children—as such perceptions can be related to each other and can potentially be associated with clinical outcomes. Similarly, doctors should always be aware of how much certain clinical outcomes (e.g., HbA1c values) might significantly affect the course of their relationship with their patients.

A person-centered approach that takes into account psychological issues—such as parents’ and children’s expectations, beliefs, and perspectives, as well as their reciprocal influence—along with the contents and events that come up during a diabetes consultation seems to be crucial to positive health outcomes in diabetes care [58, 59].

## Supplementary Information

Below is the link to the electronic supplementary material.

Supplementary file1 (DOCX 86 KB)
