# Supplementary material for: Doctor-Patient Relationship in Synchronous/Real-time Video-Consultations and In-Person Visits: An Investigation of the Perceptions of Young People with Type 1 Diabetes and Their Parents During the COVID-19 Pandemic
Source: Int J Behav Med. 2022 Jan 25;29(5):638–47. doi: 10.1007/s12529-021-10047-5 (PMC8788397; doi:10.1007/s12529-021-10047-5)
Supplement: Supplementary file 1 — Supplementary file1 (DOCX 86 KB) [file 12529_2021_10047_MOESM1_ESM.docx]

Supplemental Table 4 Summary of linear regression analyses of variables in video consultations^a^ and in-person visits that predict parents’ satisfaction with care (CASC)

|  | Video - consultation (N=216) | | | |  | In person (N=305) | | | |
| --- | --- | --- | --- | --- | --- | --- | --- | --- | --- |
| Variables | B | [95% CI] | 𝛽 | *p* |  | B | [95% CI] | 𝛽 | *p* |
|  |  |  |  |  |  |  |  |  |  |
| **CASC Availability** |  |  |  |  |  |  |  |  |  |
| Step 1 |  |  |  |  |  |  |  |  |  |
| Sex | .103 | [-.207, .414] | .053 | .512 |  | .019 | [-.259, .298] | .010 | .891 |
| Age | -.015 | [-.076, .046] | -.040. | .632 |  | .029 | [-.032, .090] | .074 | .349 |
| Duration of illness | -.039 | [-.079, .001] | -.162 | .054 |  | -.010 | [-.048, .029] | -.039 | .616 |
| zBMI | .036 | [-.089, .161] | .046 | .572 |  | -.016 | [-.153, .121] | -.017 | .822 |
|  |  |  |  |  |  |  |  |  |  |
| Step 2 |  |  |  |  |  |  |  |  |  |
| Sex | .104 | [-.208, .415] | .053 | .513 |  | .040 | [-.242, .321] | .021 | .781 |
| Age | -.015 | [-.077, .046] | -.041 | .622 |  | .030 | [-.031, .091] | .078 | .326 |
| Duration of illness | -.040 | [-.080, .001] | -.165 | .054 |  | -.012 | [-.051, .026] | -.050 | .528 |
| zBMI | .034 | [-.092, .161] | .044 | .593 |  | -.021 | [-.159, .116] | -.023 | .760 |
| HbA1c | .015 | [-.143, .172] | .015 | .854 |  | .052 | [-.046, .150] | .080 | .298 |
|  |  |  |  |  |  |  |  |  |  |
| Step 3 |  |  |  |  |  |  |  |  |  |
| Sex | .167 | [.-.095, .428] | .086 | .210 |  | -.015 | [-.238, .208] | -.008 | .893 |
| Age | -.025 | [-.076, .027] | -.066 | .348 |  | .013 | [-.035, .062] | .034 | .587 |
| Duration of illness | -.022 | [-.056, .012] | -.090 | .210 |  | .006 | [-.025, .037] | .023 | .711 |
| zBMI | .045 | [-.061, .151] | .057 | .406 |  | .029 | [-.080, .138] | .032 | .603 |
| HbA1c | .004 | [-.128, .136] | .004 | .952 |  | .072 | [-.006, .150] | .111 | .070 |
| JSPPPE-physician’s empathy | .085 | [.064, .106] | .547 | .000 |  | .080 | [.065, .096] | .619 | .000 |
|  |  |  |  |  |  |  |  |  |  |
|  |  |  |  |  |  |  |  |  |  |
| **CASC interpersonal skills** |  |  |  |  |  |  |  |  |  |
| Step 1 |  |  |  |  |  |  |  |  |  |
| Sex | .026 | [-.279, .332] | .014 | .865 |  | .044 | [-.234, .321] | .023 | .755 |
| Age | -.009 | [-.069, .051] | -.024 | .773 |  | .045 | [-.016, .106] | .115 | .114 |
| Duration of illness | -.035 | [-.074, .004] | -.147 | .082 |  | -.030 | [-.068, .008] | -.121 | .122 |
| zBMI | .042 | [-.081, .165] | .055 | .497 |  | -.048 | [-.184, .089] | -.052 | .490 |
|  |  |  |  |  |  |  |  |  |  |
| Step 2 |  |  |  |  |  |  |  |  |  |
| Sex | .027 | [-.279, .333] | .014 | .863 |  | .064 | [-.216, .344] | .034 | .654 |
| Age | -.010 | [-.071, .050] | -.028 | .737 |  | .047 | [-.014, .107] | .118 | .132 |
| Duration of illness | -.036 | [-.076, .003] | -.154 | .072 |  | -.033 | [-.071, .006] | -.132 | .096 |
| zBMI | .038 | [-.086, .162] | .049 | .546 |  | -.053 | [-.190, .083] | -.058 | .443 |
| HbA1c | .042 | [-.113, .196] | .044 | .593 |  | .051 | [-.047, .149] | .078 | .307 |
|  |  |  |  |  |  |  |  |  |  |
| Step 3 |  |  |  |  |  |  |  |  |  |
| Sex | .101 | [-.131, .333] | .053 | .392 |  | .003 | [-.203, .208] | .002 | .978 |
| Age | -.021 | [-.067, .025] | -.058 | .363 |  | .028 | [-.017, .072] | .070 | .223 |
| Duration of illness | -.015 | [-.046, .015] | -.064 | .322 |  | -.013 | [-.041, .016] | -.050 | .386 |
| zBMI | .050 | [-.044, .144] | .065 | .293 |  | .002 | [-.098, .103] | .002 | .965 |
| HbA1c | .029 | [-.088, .146] | .031 | .621 |  | .072 | [.001, .145] | .111 | .070 |
| JSPPPE-physician’s empathy | 1.00 | [.081, .118] | .655 | .000 |  | .089 | [.074, .103] | .683 | .000 |
|  |  |  |  |  |  |  |  |  |  |
|  |  |  |  |  |  |  |  |  |  |
| **CASC Technical skills** |  |  |  |  |  |  |  |  |  |
| Step 1 |  |  |  |  |  |  |  |  |  |
| Sex | .043 | [-.242, 328] | .025 | .765 |  | .048 | [-.193, .290] | .030 | .694 |
| Age | -.012 | [-.068, .044] | -.036 | .667 |  | .011 | [-.042, .064] | .031 | .689 |
| Duration of illness | -.024 | [-.061, .012] | -.112 | .187 |  | -.008 | [-.041, .026] | -.035 | .656 |
| zBMI | -.002 | [-.117, .113] | -.003 | .974 |  | -.092 | [-.210, .027] | -.115 | .130 |
|  |  |  |  |  |  |  |  |  |  |
| Step 2 |  |  |  |  |  |  |  |  |  |
| Sex | .043 | [-.243, .329] | .024 | .766 |  | .056 | [-.188, .301] | .035 | .650 |
| Age | -.012 | [-.068, .045] | -.035 | .678 |  | .011 | [-.042, .064] | .033 | .675 |
| Duration of illness | -.024 | [-.061, .013] | -.110 | .200 |  | -.009 | [-.042, .025] | -.040 | .615 |
| zBMI | -.001 | [-.117, .115] | -.001 | .978 |  | -.094 | [-.213, .025] | -.118 | .122 |
| HbA1c | -.009 | [-.153, .135] | -.010 | .904 |  | .021 | [-.065, .106] | .037 | .630 |
|  |  |  |  |  |  |  |  |  |  |
| Step 3 |  |  |  |  |  |  |  |  |  |
| Sex | .105 | [-.126, .337] | .060 | .369 |  | .008 | [-.183, .199] | .005 | .937 |
| Age | -.021 | [-.067, .025] | -.062 | .365 |  | -.004 | [-.045, .038] | -.011 | .854 |
| Duration of illness | -.006 | [-.036, .024] | -.029 | .679 |  | .008 | [-.019, .034] | .035 | .572 |
| zBMI | .009 | [-.084, .103] | .013 | .844 |  | -.049 | [-.143, .044] | -.062 | .299 |
| HbA1c | -.019 | [-.136, .097] | .022 | .744 |  | .039 | [-.028, .105] | .069 | .256 |
| JSPPPE-physician’s empathy | .084 | [.066, .102] | .595 | .000 |  | .071 | [.058, .084] | .631 | .000 |
|  |  |  |  |  |  |  |  |  |  |
|  |  |  |  |  |  |  |  |  |  |
| **CASC Information provision** |  |  |  |  |  |  |  |  |  |
| Step 1 |  |  |  |  |  |  |  |  |  |
| Sex | .016 | [-.281, .312] | .008 | .918 |  | .058 | [-.192, .309] | .035 | .647 |
| Age | -.024 | [-.083, .034] | -.069 | .412 |  | .040 | [-.015, .095] | .113 | .150 |
| Duration of illness | -.023 | [-.060, .015] | -.098 | .243 |  | -.006 | [-.041, .029] | -.026 | .735 |
| zBMI | .000 | [-.120, .119] | .000 | .996 |  | -.070 | [-.193, .054] | -.084 | .266 |
|  |  |  |  |  |  |  |  |  |  |
| Step 2 |  |  |  |  |  |  |  |  |  |
| Sex | .016 | [-.282, .313] | .009 | .917 |  | .075 | [-.178, .328] | .045 | .558 |
| Age | -.025 | [-.084, .034] | -.071 | .402 |  | .041 | [-.014, .096] | .116 | .139 |
| Duration of illness | -.023 | [-.062, .015] | -.102 | .235 |  | -.008 | [-.043, .027] | -.036 | .644 |
| zBMI | -.002 | [-.123, .119] | -.003 | .970 |  | -.074 | [-.198, .049] | -.090 | .236 |
| HbA1c | .019 | [-.131, .169] | .021 | .799 |  | .044 | [-.044, .133] | .075 | .327 |
|  |  |  |  |  |  |  |  |  |  |
| Step 3 |  |  |  |  |  |  |  |  |  |
| Sex | .073 | [-.181, .328] | .040 | .570 |  | .031 | [-.181, .244] | .019 | .771 |
| Age | -.033 | [-.084, .017] | -.095 | .190 |  | .028 | [-.019, .074] | .078 | .239 |
| Duration of illness | -.007 | [-.040, .026] | -.030 | .684 |  | .006 | [-.023, .036] | .029 | .666 |
| zBMI | .007 | [-.096, .110] | .010 | .890 |  | -.034 | [-.138, .070] | -.041 | .517 |
| HbA1c | .010 | [-.118, .138] | .011 | .882 |  | .060 | [-.014, .134] | .103 | .113 |
| JSPPPE-physician’s empathy | .078 | [.057, .098] | .527 | .000 |  | .064 | [.049, .079] | .548 | .000 |
|  |  |  |  |  |  |  |  |  |  |

^a^values as estimated in participants using CGM/FGM (N=216)

Abbreviations: zBMI=standardized body mass index; HbA1c=glycated hemoglobin; CASC=Comprehensive assessment of satisfaction with care; JSPPPE= Jefferson scale of patient perceptions of physician empathy.

T1D video-consultation visit: CASC Availability R^2^ = .035 for Step 1 (p = .246); ΔR^2^ = .000 for Step 2 (p = .364); ΔR^2^ = .292 for Step 3 (p = .000); tot R^2^ = .327; CASC Interpersonal skills R^2^ =.027 for Step 1 (p =.381); ΔR^2^ = .002 for Step 2 (p=.485); ΔR^2^ = .418 for Step 3 (p =.000); tot R^2^ =.447; CASC Technical skills R^2^ =.016 for Step 1 (p =.655); ΔR^2^ = .000 for Step 2 (p=.784); ΔR^2^ = .345 for Step 3 (p =.000); tot R^2^ =.361; CASC Information provision R^2^ =.018 for Step 1 (p =.594); ΔR^2^ = .000 for Step 2 (p=.724); ΔR^2^ = .271 for Step 3 (p =.000); tot R^2^ =.289. The collinearity statistics of the predictors showed tolerance values in the range of .872–.976, thus excluding multicollinearity.

T1D in person visit: CASC Availability R^2^ = .006 for Step 1 (p = .902); ΔR^2^ = .006 for Step 2 (p = .829); ΔR^2^ = .372 for Step 3 (p = .000); tot R^2^= .384; CASC Interpersonal skills R^2^ =.024 for Step 1 (p =.380); ΔR^2^ = .006 for Step 2 (p=.388); ΔR^2^ = .453 for Step 3 (p =.000); tot R^2^ =.482; CASC Technical skills R^2^ =.015 for Step 1 (p =.604); ΔR^2^ = .001 for Step 2 (p=.707); ΔR^2^ = .386 for Step 3 (p =.000); tot R^2^ =.403; CASC Information provision R^2^ =.021 for Step 1 (p =.442); ΔR^2^ = .005 for Step 2 (p=.453); ΔR^2^ = .291 for Step 3 (p =.000); tot R^2^ =.318. The collinearity statistics of the predictors showed tolerance values in the range of .898–.994, thus excluding multicollinearity.

Supplemental Table 5 Summary of linear regression analyses of variables in video consultations^a^ and in-person visits that predict parents’ perception of alliance in the doctor-patient relationship (WAI-S)

|  | Video - consultation (N=216) | | | |  | In person (N=305) | | | |
| --- | --- | --- | --- | --- | --- | --- | --- | --- | --- |
| Variables | B | [95% CI] | 𝛽 | *p* |  | B | [95% CI] | 𝛽 | *p* |
|  |  |  |  |  |  |  |  |  |  |
| **WAI-S** **Agreement on goals** |  |  |  |  |  |  |  |  |  |
| Step 1 |  |  |  |  |  |  |  |  |  |
| Sex | .281 | [-1.21, 1.77] | .030 | .711 |  | 1.575 | [.108, 3.04] | .157 | .036 |
| Age | .108 | [-.186, .403] | .061 | .467 |  | .285 | [-.036, .606] | .135 | .081 |
| Duration of illness | -.143 | [-.335, .048] | -.124 | .141 |  | -.165 | [-.367, .038] | -.123 | .111 |
| zBMI | -.039 | [-.641, .564] | -.010 | .900 |  | -.222 | [-.943, .499] | -.045 | .545 |
|  |  |  |  |  |  |  |  |  |  |
| Step 2 |  |  |  |  |  |  |  |  |  |
| Sex | .286 | [-1.21, 1.78] | .031 | .706 |  | 1.331 | [-.130, 2.79] | .133 | .074 |
| Age | .089 | [-.205, .304] | .050 | .550 |  | .268 | [-.049, .585] | .127 | .097 |
| Duration of illness | -.164 | [-.357, .029] | -.143 | .095 |  | -.133 | [-.334, .069] | -.099 | .196 |
| zBMI | -.093 | [-.699, .513] | -.025 | .762 |  | -.154 | [-.867, .560] | -.031 | .672 |
| HbA1c | .525 | [-.228, 1.28] | .114 | .170 |  | -.628 | [-1.14, -.117] | -.180 | .016 |
|  |  |  |  |  |  |  |  |  |  |
| Step 3 |  |  |  |  |  |  |  |  |  |
| Sex | .470 | [-.943, 1.88] | .051 | .512 |  | 1.179 | [-.207, 2.56] | .118 | .095 |
| Age | .062 | [-.216, .341] | .035 | .659 |  | .221 | [-.080, .522] | .105 | .149 |
| Duration of illness | -.112 | [-.296, .073] | -.097 | .233 |  | -.082 | [-.274, .110] | -.061 | .401 |
| zBMI | -.062 | [-.636, .511] | -.017 | .830 |  | -.015 | [-.694, .664] | -.003 | .966 |
| HbA1c | .494 | [-.218, 1,21] | .107 | .172 |  | -.573 | [-1.06, -.088] | -.164 | .021 |
| JSPPPE-physician’s empathy | .247 | [.135, .360] | .334 | .000 |  | .221 | [.125, .318] | .317 | .000 |
|  |  |  |  |  |  |  |  |  |  |
|  |  |  |  |  |  |  |  |  |  |
| **WAI-S** **Agreement on tasks** |  |  |  |  |  |  |  |  |  |
| Step 1 |  |  |  |  |  |  |  |  |  |
| Sex | -.162 | [-1.28, .954] | -.023 | .774 |  | .430 | [-.589, 1.45] | .062 | .406 |
| Age | .033 | [-.187, .252] | .025 | .768 |  | .217 | [-.005, .440] | .148 | .056 |
| Duration of illness | -.107 | [-.250, .036] | -.124 | .141 |  | -.169 | [-.310, -.029] | -.182 | .019 |
| zBMI | -.217 | [-.666, .232] | -.077 | .342 |  | -.319 | [-.820, .182] | -.093 | .210 |
|  |  |  |  |  |  |  |  |  |  |
| Step 2 |  |  |  |  |  |  |  |  |  |
| Sex | -.161 | [-1.28, .958] | .023 | .776 |  | .354 | [-.674, 1.38] | .051 | .498 |
| Age | .028 | [-.193, .249] | .021 | .804 |  | .212 | [-.011, .435] | .145 | .062 |
| Duration of illness | -.113 | [-.258, .032] | -.131 | .127 |  | -.159 | [-.301, -.018] | -.172 | .028 |
| zBMI | -.231 | [-.686, .223] | -.083 | .316 |  | -.298 | [-.800, .204] | -.087 | .243 |
| HbA1c | .139 | [-.426, .703] | .040 | .628 |  | -.195 | [-.555, .164] | -.081 | .285 |
|  |  |  |  |  |  |  |  |  |  |
| Step 3 |  |  |  |  |  |  |  |  |  |
| Sex | .116 | [-.718, .950] | .017 | .784 |  | .146 | [-.651, .943] | .021 | .718 |
| Age | -.013 | [-.177, .152] | -.010 | .879 |  | .148 | [-.026, .321] | .100 | .094 |
| Duration of illness | -.034 | [-.142, .075] | -.039 | .541 |  | -.090 | [-.201, .020] | -.097 | .109 |
| zBMI | -.186 | [-.524, .152] | -.066 | .280 |  | -.108 | [-.499, .282] | -.032 | .585 |
| HbA1c | .092 | [-.328, .512] | .027 | .665 |  | -.120 | [-.399, .159] | -.049 | .398 |
| JSPPPE-physician’s empathy | .373 | [.306, .439] | .671 | .000 |  | .303 | [.248, .358] | .626 | .000 |
|  |  |  |  |  |  |  |  |  |  |
|  |  |  |  |  |  |  |  |  |  |
| **WAI-S** **Bond** |  |  |  |  |  |  |  |  |  |
| Step 1 |  |  |  |  |  |  |  |  |  |
| Sex | -.357 | [-1.74, 1.03] | -.042 | .611 |  | .472 | [-.731, 1.67] | .058 | .440 |
| Age | .023 | [-.249, .295] | .014 | .870 |  | .196 | [-.067, .460] | .114 | .142 |
| Duration of illness | -.073 | [-.250, .104] | -.069 | .418 |  | -.206 | [-.372, -.040] | -.189 | .015 |
| zBMI | -.164 | [-.722, .393] | -.048 | .561 |  | -.210 | [-.801, .381] | -.052 | .484 |
|  |  |  |  |  |  |  |  |  |  |
| Step 2 |  |  |  |  |  |  |  |  |  |
| Sex | -.357 | [-1.75, 1.03] | -.042 | .613 |  | .295 | [-.907, 1.49] | .036 | .628 |
| Age | .022 | [-.252, .296] | .013 | .873 |  | .184 | [-.077, .445] | .107 | .165 |
| Duration of illness | -.073 | [-.253, .106] | -.069 | .422 |  | -.183 | [-.349, -.017] | -.168 | .031 |
| zBMI | -.166 | [-.730, .398] | -.048 | .563 |  | -.161 | [-.748, .426] | -.040 | .590 |
| HbA1c | .014 | [-.687, .714] | .003 | .969 |  | -.454 | [-.875, -.034] | -.160 | .034 |
|  |  |  |  |  |  |  |  |  |  |
| Step 3 |  |  |  |  |  |  |  |  |  |
| Sex | .006 | [-.980, .992] | .001 | .991 |  | .026 | [-.829, .880] | .003 | .953 |
| Age | -.031 | [-.225, .164] | -.019 | .754 |  | .100 | [-.085, .286] | .058 | .288 |
| Duration of illness | .030 | [-.099, .158] | .028 | .646 |  | -.093 | [-.212, .025] | -.085 | .123 |
| zBMI | -.106 | [-.506, .294] | -.031 | .602 |  | .086 | [-.332, .504] | .021 | .686 |
| HbA1c | -.047 | [-.544, .449] | -.011 | .851 |  | -.356 | [-.655, -.057] | -.125 | .020 |
| JSPPPE-physician’s empathy | .488 | [.401, .566] | .713 | .000 |  | .394 | [.334, .453] | .692 | .000 |
|  |  |  |  |  |  |  |  |  |  |

^a^values as estimated in participants using CGM/FGM (N=216)

Abbreviations: zBMI=standardized body mass index; HbA1c=glycated hemoglobin; WAI-S= Working Alliance Inventory short form; JSPPPE= Jefferson scale of patient perceptions of physician empathy.

T1D video-consultation visit: WAI-S Agreement on goals R^2^ = .015 for Step 1 (p = .670); ΔR^2^ = .012 for Step 2 (p = .513); ΔR^2^ = .109 for Step 3 (p = .001); tot R^2^ = .136; Agreement on tasks R^2^ = .023 for Step 1 (p = .468); ΔR^2^ = .002 for Step 2 (p = .579); ΔR^2^ = .439 for Step 3 (p = .000); tot R^2^ = .464; Bond R^2^ = .010 for Step 1 (p = .819); ΔR^2^ = .000 for Step 2 (p = .908); ΔR^2^ = .496 for Step 3 (p = .000); tot R^2^ = .506. The collinearity statistics of the predictors showed tolerance values in the range of .872–.976, thus excluding multicollinearity.

T1D in person visit: WAI-S Agreement on goals R^2^ = .052 for Step 1 (p = .052); ΔR^2^ = .031 for Step 2 (p = .010); ΔR^2^ = .098 for Step 3 (p = .000); tot R^2^= .181; Agreement on tasks R^2^ = .053 for Step 1 (p = .050); ΔR^2^ = .006 for Step 2 (p = .059); ΔR^2^ = .380 for Step 3 (p = .000); tot R^2^= .439; Bond R^2^ = .043 for Step 1 (p = .106); ΔR^2^ = .025 for Step 2 (p = .033); ΔR^2^ = .466 for Step 3 (p = .000); tot R^2^ = .533. The collinearity statistics of the predictors showed tolerance values in the range of .898–.994, thus excluding multicollinearity.

Supplemental Table 6 Summary of linear regression analyses of variables in video consultations^a^ and in-person visits that predict patients’ perceptions of physician empathy (JSPPPE)

|  | Video - consultation (N=216) | | | |  | In person (N=305) | | | |
| --- | --- | --- | --- | --- | --- | --- | --- | --- | --- |
| Variables | B | [95% CI] | 𝛽 | *p* |  | B | [95% CI] | 𝛽 | *p* |
|  |  |  |  |  |  |  |  |  |  |
| Step 1 |  |  |  |  |  |  |  |  |  |
| Sex | -.744 | [-2.75, 1.26] | -.060 | .465 |  | .782 | [-1.35, 2.91] | .054 | .470 |
| Age | .113 | [-.282, .508] | .047 | .572 |  | .220 | [-.246, .687] | .073 | .353 |
| Duration of illness | -.207 | [-.464, .050] | -.133 | .114 |  | -.241 | [-.535, .054] | -.126 | .108 |
| zBMI | -.110 | [-.919, .699] | -.022 | .789 |  | -.654 | [-1.70, .394] | -.092 | .220 |
|  |  |  |  |  |  |  |  |  |  |
| Step 2 |  |  |  |  |  |  |  |  |  |
| Sex | -.743 | [-2.76, 1.27] | -.060 | .467 |  | .685 | [-1.47, 2.84] | .048 | .531 |
| Age | .109 | [-.289, .506] | .045 | .590 |  | .213 | [-.254, .681] | .070 | .369 |
| Duration of illness | -.212 | [-.473, .049] | -.136 | .111 |  | -.228 | [-.525, .069] | -.119 | .132 |
| zBMI | -.123 | [-.941, .696] | -.024 | .768 |  | -.626 | [-1.68, .472] | -.089 | .242 |
| HbA1c | .125 | [-.891, 1.14] | .020 | .808 |  | -.249 | [-1.01, .505] | .050 | .515 |
|  |  |  |  |  |  |  |  |  |  |
| Step 3 |  |  |  |  |  |  |  |  |  |
| Sex | -.534 | [-1.83, .765] | -.043 | .418 |  | .136 | [-1.27, 1.54] | .009 | .848 |
| Age | .078 | [-.178, .334] | .033 | .548 |  | -.080 | [-.319, .231] | -.026 | .612 |
| Duration of illness | -.054 | [-.226, .118] | -.035 | .533 |  | .051 | [-.149, .251] | .027 | .613 |
| zBMI | -.113 | [-.646, .421] | -.022 | .677 |  | -.325 | [-1.02, .365] | -.046 | .353 |
| HbA1c | -.035 | [-.694, .624] | -.006 | .916 |  | .060 | [-.454, .573] | .012 | .819 |
| CASC Availability | -.296 | [-1.94, 1.35] | .046 | .723 |  | -.577 | [-2.18, 1.03] | -.075 | .478 |
| CASC Interpersonal skills | 3.82 | [2.09, 5.55] | .581 | .000 |  | 4.04 | [2.19, 5.90] | .526 | .000 |
| CASC Technical skills | .120 | [-2.02, 2.26] | .017 | .912 |  | .679 | [-1.26, 2.62] | .076 | .490 |
| CASC Information provision | -1.83 | [-3.54,-.124] | -.270 | .036 |  | -1.508 | [-3.24, .225] | -.177 | .088 |
| WAI-S Agreement on goals | .019 | [-.151, .189] | .014 | .822 |  | .116 | [-.039, .271] | .081 | .140 |
| WAI-S Agreement on tasks | .162 | [-.246, .569] | .090 | .434 |  | .004 | [-.411, .420] | .002 | .983 |
| WAI-S Bond | .663 | [.381, .946] | .454 | .000 |  | .789 | [.461, 1.12] | .449 | .000 |
|  |  |  |  |  |  |  |  |  |  |

^a^values as estimated in participants using CGM/FGM (N=216)

Abbreviations: zBMI=standardized body mass index; HbA1c=glycated hemoglobin; CASC=Comprehensive assessment of satisfaction with care; WAI-S= Working Alliance Inventory short form; JSPPPE= Jefferson scale of patient perceptions of physician empathy.

T1D video-consultation visit: JSPPPE R^2^ = .023 for Step 1 (p = .457); ΔR^2^ = .000 for Step 2 (p = .595); ΔR^2^ = .597 for Step 3 (p = .000); tot R^2^ = .621. The collinearity statistics of the predictors showed tolerance values in the range of 0.112–0.976, thus ruling out multicollinearity.

T1D in person visit: JSPPPE R^2^ = .027 for Step 1 (p = .312); ΔR^2^ = .002 for Step 2 (p = .394); ΔR^2^ = .584 for Step 3 (p = .000); tot R^2^ = .614. The collinearity statistics of the predictors showed tolerance values in the range of .156–.994, thus excluding multicollinearity.
